# Supplementary material for: QUICK: Quality and Usability Investigation and Control Kit for Mass Spectrometric Data from Detection of Persistent Organic Pollutants
Source: Int J Environ Res Public Health. 2019 Oct 30;16(21):4203. doi: 10.3390/ijerph16214203 (PMC6862152; doi:10.3390/ijerph16214203)
Supplement: Supplementary file 1 [file ijerph-16-04203-s001.pdf]

Table S1: Congener Number and its name.

Congener #	Congener Name
1	1234678-HpCDD
2	1234678-HpCDF
3	123478-HxCDD
4	123478-HxCDF
5	1234789-HpCDF
6	123678-HxCDD
7	123678-HxCDF
8	12378-PeCDD
9	12378-PeCDF
10	123789-HxCDD
11	123789-HxCDF
12	12C-126-PCB
13	12C-169-PCB
14	12C-77-PCB
15	12C-81-PCB
16	13C-1234678-HpCDD
17	13C-1234678-HpCDF
18	13C-123478-HxCDD
19	13C-123478-HxCDF
20	13C-1234789-HpCDF
21	13C-123678-HxCDD
22	13C-123678-HxCDF
23	13C-12378-PeCDD
24	13C-12378-PeCDF
25	13C-123789-HxCDF
26	13C-126-PCB
27	13C-169-PCB
28	13C-234678-HxCDF
29	13C-23478-PeCDF
30	13C-2378-TCDD
31	13C-2378-TCDF
32	13C-77-PCB
33	13C-81-PCB
34	13C-OCDD
35	234678-HxCDF
36	23478-PeCDF
37	2378-TCDD
38	2378-TCDF
39	OCDD
40	OCDF

C-13 congeners are Internal and Recovery Standards

Figure Captions

Figure S1: Trendplot for 13C-OCDD in Standard Data. Calculated concentrations are plotted at y-axis. Sample names are given in the x-axis. The mean and standard deviation of calculated concentration are calculated from all the data except the rightmost one in the plot. The solid red

line at around 20.5 represent the mean value of the calculated concentrations. The red region spans from 2SD to 3SD from the mean value. The rightmost point represents 13C-OCDD from the standard sample P180629-CS3A.

Figure S2: Trendplot for 1234789-HpCDF in Spike Data. Calculated concentrations are plotted at y-axis. Sample names are given in the x-axis. The mean and standard deviation of calculated concentration are calculated from all the data except the rightmost one in the plot. The solid red line at around 4.8 represent the mean value of the calculated concentrations. The red region spans from 2SD to 3SD from the mean value. The rightmost point represents 1234789-HpCDF from the Spike sample XS-180905.

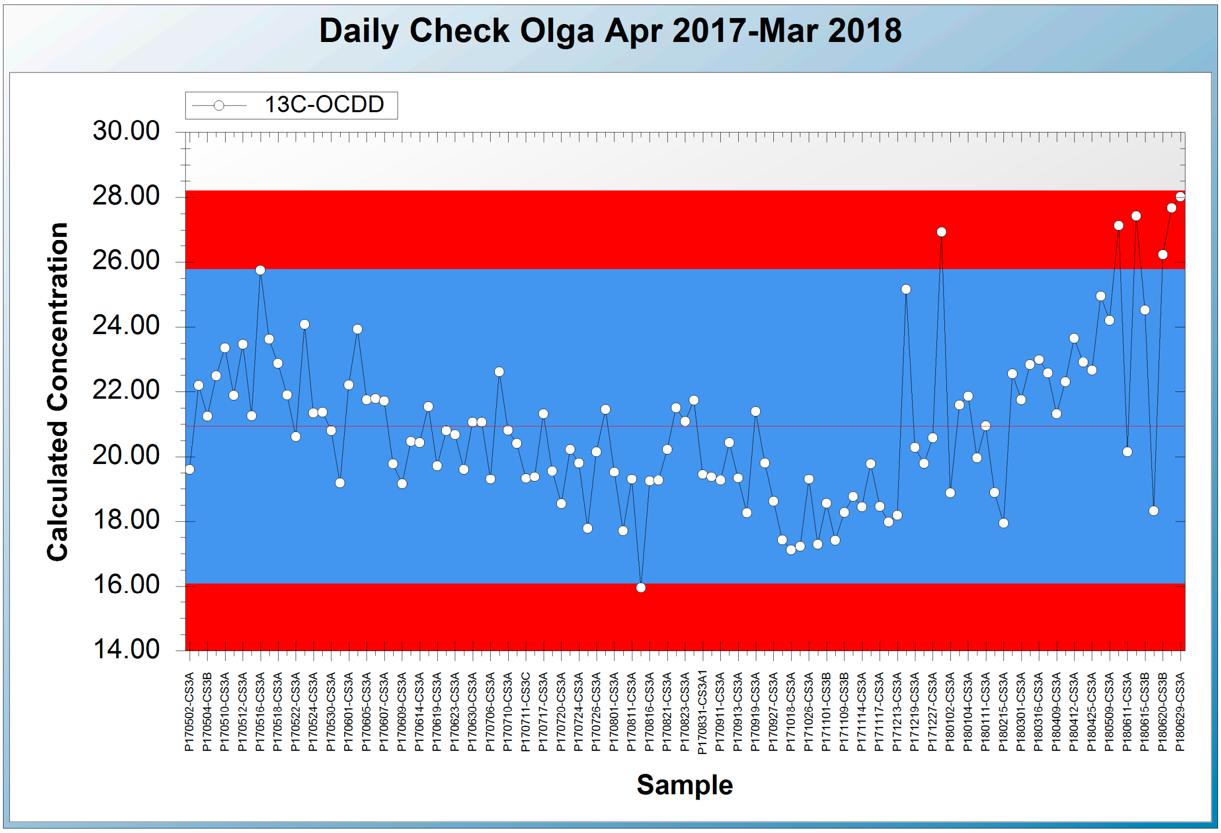

Figure S1

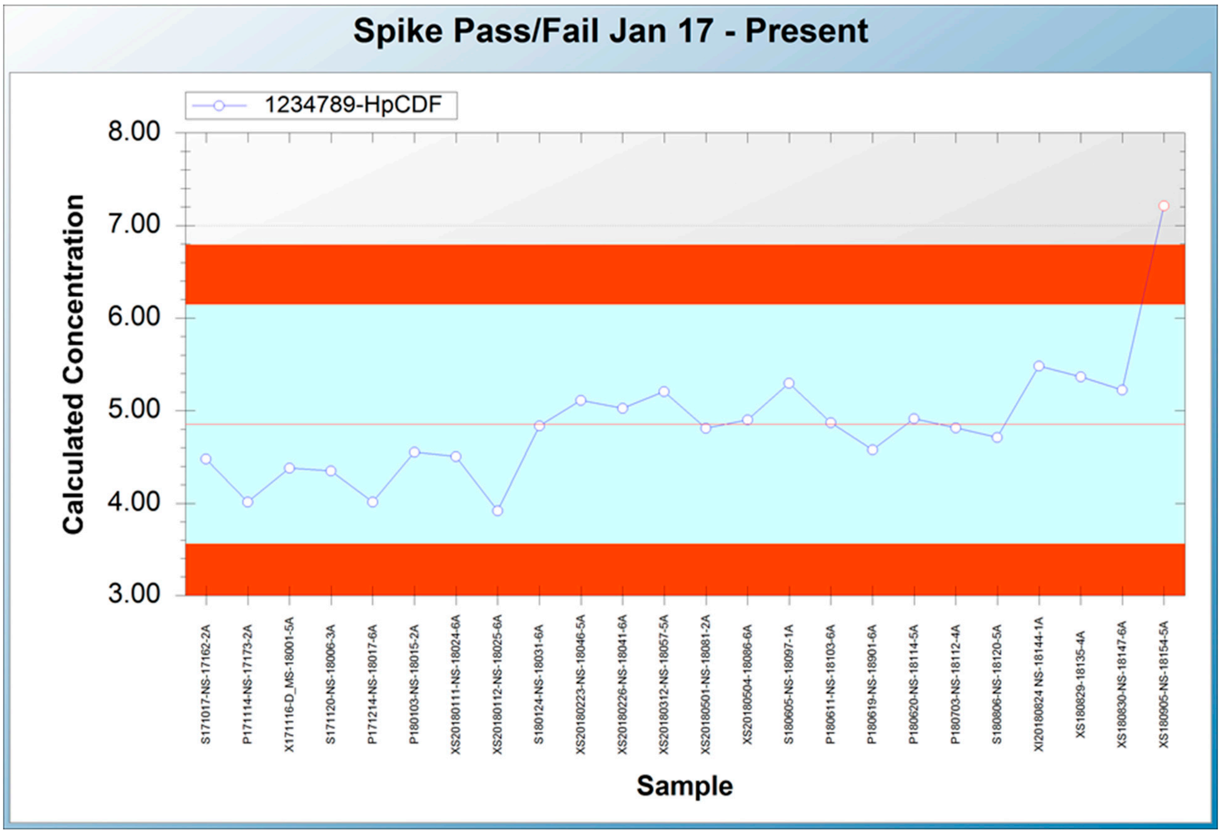
Spike Pass/Fail Jan 17 - Present
1234789-HpCDF
Calculated Concentration
8.00
7.00
6.00
5.00
4.00
3.00
S171017-NS-17162-2A
P171114-NS-17173-2A
X171116-D_MS-18001-5A
S171120-NS-18006-3A
P171214-NS-18017-6A
P180103-NS-18015-2A
XS20180111-NS-18024-6A
XS20180112-NS-18025-6A
S180124-NS-18031-6A
XS20180223-NS-18046-5A
XS20180226-NS-18041-6A
XS20180312-NS-18057-5A
XS20180501-NS-18081-2A
XS20180504-18086-6A
S180605-NS-18097-1A
P180611-NS-18103-6A
P180619-NS-18901-6A
P180620-NS-18114-5A
P180703-NS-18112-4A
S180806-NS-18120-5A
XI20180824 NS-18144-1A
XS180829-18135-4A
XS180830-NS-18147-6A
XS180905-NS-18154-5A
Sample

Figure S2
